# A sharp increase in the number of COVID-19 cases and case fatality rates after lifting the lockdown in Kurdistan region of Iraq

**DOI:** 10.1016/j.amsu.2020.07.030

**Published:** 2020-07-24

**Authors:** Nawfal R. Hussein, Ibrahim A. Naqid, Zana Sidiq M. Saleem, Lokman A. Almizori, Dildar H. Musa, Nashwan Ibrahim

**Affiliations:** a College of Medicine, University of Zakho, Kurdistan Region, Iraq; b College of Medicine, University of Duhok, Kurdistan Region, Iraq; c Department of Medicine, Azadi Teaching Hospital, Duhok, Kurdistan Region, Iraq

**Keywords:** COVID-19 cases, Case fatality rates, Lockdown, Kurdistan region, Iraq

## Abstract

With the appearance of first cases of Coronavirus disease (COVID-19), strict control measures were implemented in the Kurdistan Region of Iraq to combat the infection. These measures included the closure of schools and universities, the closure of borders and airports, cancellation of public and religious gatherings, and mandatory quarantine for persons returning from traveling abroad. Such measures have played a major role in the control of COVID-19 spread. However, due to social and economic pressures, the government relaxed the lockdown. After relaxing the measures, a sharp increase in the number of patients was noticed. Besides, there was a significant increase in the number of symptomatic patients and the case fatality rate was doubled. In addition, the outbreak and outbreak response led to the loss of trust and a breakdown in relations between the society and local authority. To minimize the consequences for population health, local authority should have a plan that balances between health imperatives and socioeconomic imperatives. Finally, to be successful in controlling the infection, the government must rebuild public trust in the handling of COVID-19 outbreak and compensate people for lost earnings.

Dear Editor:

SARS-CoV-2 is a novel virus causing the coronavirus disease (COVID-19), which is an emerging respiratory disease that was identified in November 2019 in Wuhan, China [1]. It has spread rapidly worldwide. The World Health Organization declared COVID-19 a global pandemic on March 11, 2020 [2]. The first case of COVID-19 was diagnosed in Iraq on the February 22, 2020. With the appearance of first cases in the country, Kurdistan Regional Government, the northern Region of Iraq, took strict measures to control the infection. These measures included cancellation of gathering and religious rituals, closing schools and education institutes and closing airports and boarders [3]. Then, state-imposed community-wide containment was declared [3,4]. The Ministry of Health in Kurdistan Region imposed strict regulations that all PCR-positive patients must be admitted to COVID centers regardless the presence of symptoms [5]. COVID centers are dedicated centers for the treatment of COVID-19 patients only. Additionally, all subjects who returned back from abroad, other regions of Iraq, or come in contact with a PCR-positive patient were quarantined for 14 days [5]. In the quarantine, PCR test was performed at the beginning and at the end of quarantine period. The containment measures started on the March 1, 2020 and continued till the May 21, 2020. Then, political necessity and mounting socioeconomic pressure demanded the start of a reopening process and sometimes at a progressively elevated pace. The considerable impact of economy and mounting pressure on the government increased the likelihood of losing control and closed the decision-making process prematurely before exploring reasonable alternatives. The lockdown was lifted and the measures were eased on the May 22, 2020. Easing the lockdown did not include any plan to protect high-risk groups including elderly, people with disabilities and people with underlying health conditions. Additionally, lifting the lockdown made a false sense of security and people started to gather again. In some cities, after cessation of cases for few days, victory against the virus was celebrated.

After relaxing the measures, a sharp increase in the number of patients was noticed (Table 1) (Fig. 1). Besides, there was a significant increase in the number of symptomatic patients after easing the lockdown and the case fatality rate was doubled (Table 1).

**Table 1:** Frequency and percentage of COVID-19 cases during and after lockdown in Kurdistan Region, Iraq (n = 2296).

Variable	During lockdown	After lockdown	Statistics test	P Value
Confirmed Cases (Average ± SD)	5.51 ± 7.89	76.83 ± 57.09	Mann-Whitney	<0.001
Death cases No. (%)	5 (1.12)	54 (2.93)	Chi square	<0.01
Symptomatic cases No. (%)	128 (14.1)	780 (85.9)	Chi square	<0.001

**Fig. 1:**
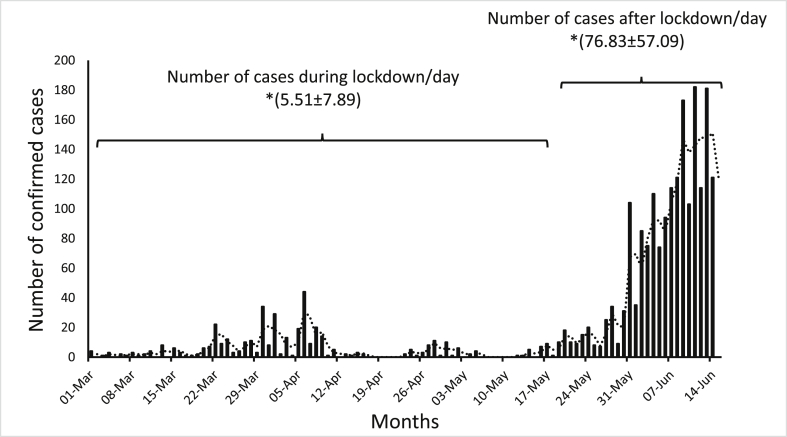
Distribution of Confirmed COVID-19 cases/day during and after lockdown in Kurdistan Region, Iraq.

Despite the sharp increase in morbidity and mortality of COVID infection, there was a perceived unwillingness of the society to follow the instructions of social distancing and infection prevention. The outbreak and outbreak response led to a profound breakdown in trust between the society and the local government. Myths that coronavirus was political game or a plot by local governments became widely-held beliefs. Coupled with the devastating socioeconomic impacts of the lockdown due to the reduction of community cohesion, loss of education, widespread loss of jobs and insecurity of food. All these factors led to the loss of trust and a breakdown in relations between the society and local authority and therefore the community was reluctant fighting the outbreak. On the other hand, local authorities are letting politics and economics drive the response at the expense of health. Such an approach is unsustainable and will not return the economy to normal. The region is going through a second destructive wave that is costing more lives. To avoid the consequences for population health, the government should have a plan that balances between health imperatives and socioeconomic imperatives. The local authorities need to rebuild public trust in the handling of COVID-19 outbreak and compensate people for lost earnings. If the people are financially compensated, it will be a big incentive to commit to the social distancing measures. Finally, the government should be transparent in how scientific advice flowed through to the prevention policy. This may repair public confidence in the local authorities handling of the crisis and prevent the resurgence of the epidemic.

## Funding/Support

No funding or support

## Ethical approval

The study was approved by the Scientific and Ethics Committee, College of Medicine, University of Zakho, Kurdistan region, Iraq.

## Authors’ contribution

We confirm that the manuscript has been contributed, reviewed and approved by all authors. We further confirm that the order of authors listed in the manuscript has been approved by all of us.

## Registration of research studies

1.Name of the registry:

2.Unique Identifying number or registration ID:

3.Hyperlink to your specific registration (must be publicly accessible and will be checked).

## Guarantor

Professor Nawfal R Hussein.

Email: nawfal.hussein@uoz.edu.krd.

Dr Ibrahim Abdulqader Naqid.

Email: Ibrahim.naqid@uoz.edu.krd.

## Provenance and peer review

Not commissioned, externally peer reviewed.

## Declaration of competing interest

The authors have declared that no competing interest exists.
